# Bilateral Pseudoexfoliation Deposits on Intraocular Lens Implants

**DOI:** 10.1155/2015/560508

**Published:** 2015-02-02

**Authors:** Elena Bonafonte Marquez, Sergio Bonafonte Royo

**Affiliations:** Centro de Oftalmología Bonafonte, 08009 Barcelona, Spain

## Abstract

We present a rare case of bilateral pseudoexfoliative deposits on both intraocular lens (IOL) implants in an 83-year-old woman with no other associated pathology, 5 years after cataract surgery. Pseudoexfoliation syndrome is the most common cause of secondary open-angle glaucoma worldwide and these deposits are usually found on the natural lens. The fact that pseudoexfoliative deposits have been found on IOL implants implies the need for a thorough examination in pseudophakic patients, for it could be the only sign of secondary glaucoma.

## 1. Introduction

Pseudoexfoliation syndrome (PEX) is widely accepted as a frequently encountered pathology that makes cataract surgery in some cases challenging. The fibrillar material can be found on all anterior segment structures bathed by the aqueous humor, but it is characterized by deposits of exfoliation material on the anterior lens capsule in a target pattern. For many years, it was thought that these deposits came from the iris pigmented epithelium, nonpigmented ciliary epithelium, or the preequatorial lens epithelium [1].

As reflected in some reports the lens is unnecessary for the production of these exfoliative deposits due to the fact that exfoliative material has been found on IOL implants, years after cataract surgery [1–5]. Most of these reports have described an exfoliative material in pseudophakic patients years after IOLs implantation, suggesting long-standing production in the absence of natural lens friction. None of these reports have described before bilateral IOL pseudoexfoliation deposits.

Recent research data suggest that exfoliation-related biochemical changes are influenced by increased oxidative stress, which, as part of a vicious circle, is enhanced by the exfoliation-induced tissue damage [6]. Local and chronic inflammation induced by the complement pathways plays a central role in the development of PEX syndrome [7].

Evidence also suggests a strong genetic component to this condition. To date a number of genes have been linked to PEX, of which LOXL1 appears to be the most relevant in many populations [8].

To our knowledge, this is the first case published having bilateral exfoliative findings on IOLs.

## 2. Case Report

**Figure 1:**
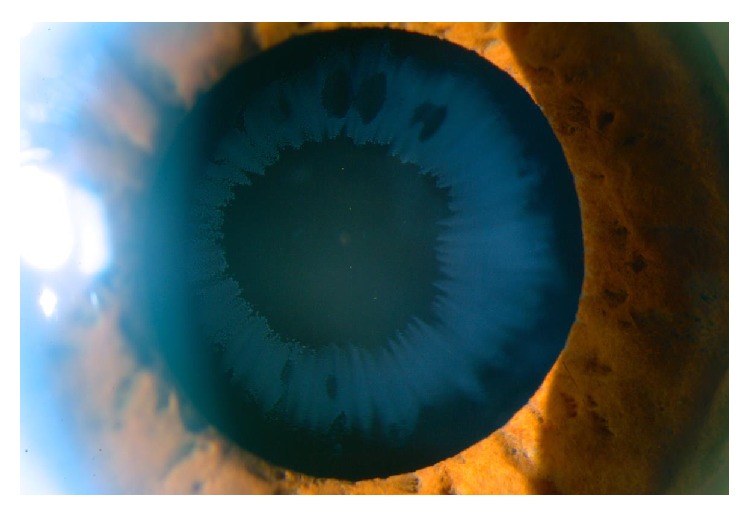
Pseudoexfoliative deposits on IOL in OD.

**Figure 2:**
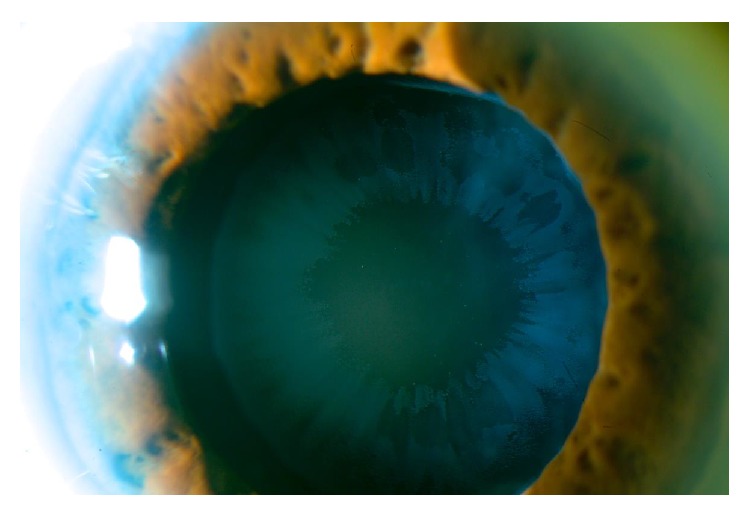
Pseudoexfoliative deposits on IOL in OS.

An 83-year-old woman with no history of glaucoma comes for a routine follow-up to the Centro de Oftalmología Bonafonte in Barcelona, Spain, presenting slightly blurred vision in both eyes for several months. As medical history, she underwent a cataract surgery with implantation of a monobloc acrylic lens from AJL Ophtalmic (Vitoria, Spain) in the capsular bag in both eyes in 2008. Her postoperative course had no complications. On examination, her visual acuity was 20/40 in both eyes with her correction and intraocular pressures (IOP) were within normal limits. Slit-lamp biomicroscopy revealed a bilateral posterior capsule opacification and pseudoexfoliative deposits on the anterior surface of both IOLs (Figures 1 and 2). No transillumination defects or glaucomatous optic nerve damage was found. Gonioscopy examination revealed an open angle with moderate segmental pigmentation of the trabecular meshwork. YAG laser capsulotomy was performed in both eyes, improving vision to 20/20.

## 3. Discussion

**Figure 3:**
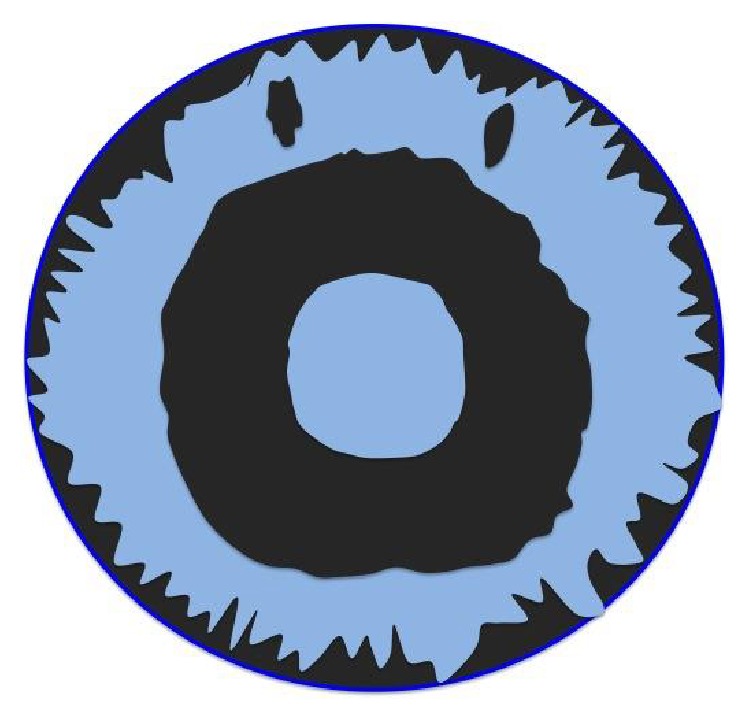
Target-like appearance pseudoexfoliation deposits on a natural lens with a central disc of pseudoexfoliation surrounded by a clear intermediate zone and the outermost deposits.

**Figure 4:**
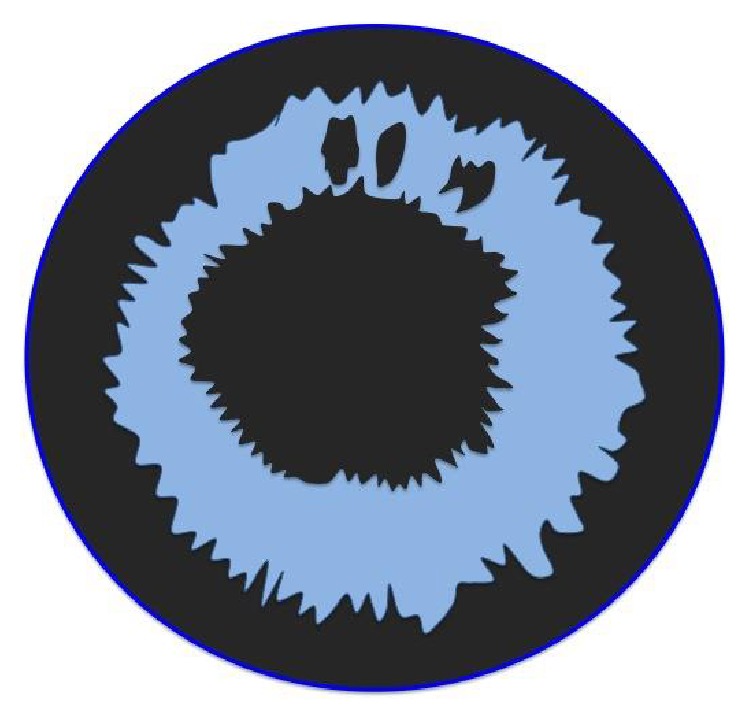
The opposite pattern observed on an IOL implant.

Pseudoexfoliation syndrome is a very frequently encountered pathology found on the natural lens of the eye appearing in a particular three-ring pattern. We can observe pseudoexfoliative deposits on the center of a natural lens and on its margin. The intermediate clear zone is caused by friction of the iris with the anterior lens capsule [1]. In this rare case, the pseudoexfoliative deposits are found on the IOL arranged in an opposite pattern to that found on a natural lens (Figures 3 and 4). The center of the IOL is free of deposits, which allows the patient to have good visual acuity. The fact that pseudoexfoliative material deposits on IOLs could be explained by long-standing production in the absence of lens friction. The presence of an IOL increases space between the iris and the IOL, prevents rubbing, and allows the buildup of exfoliative material [1]. Probably, deposits on the center and margin of the IOL get washed from the aqueous because a larger volume is able to flow in those areas, as opposed to the intermediate zone where only a small amount of aqueous humor can flow, since it is the part of the IOL that has the shortest distance to the iris.

Even though the origin of pseudoexfoliative material still remains unclear, recent publications say that exfoliation aggregates are synthesized by the nonpigmented ciliary epithelial cells, trabecular endothelial cells, vascular endothelial cells in the iris, and preequatorial lens epithelium cells. After reaching the extracellular space, exfoliation aggregates are deposited around the cells of origin but may also be passively transported by the aqueous humor and be deposited upon the zonular apparatus, anterior lens surface, and pupillary margin and within the outflow system [6].

The fact that pseudoexfoliation material can appear on the anterior surface of IOLs suggests that the material is not produced by the anterior capsule but gets deposited there from the aqueous humor.

Recently, inflammation has been gaining a very important role as a factor that can be involved in etiopathogenesis of PEX. They found IL-6 and NO levels in aqueous humor were higher in patients with PEX and pseudoexfoliative glaucoma (PEXG) than in controls. IL-6 is reported to induce an ocular inflammatory response that is often accompanied by breakdown of the blood-ocular barrier, as detected by an increased protein concentration in either the aqueous humor or vitreous fluid [7].

Variable incidence of PEX in different populations and increased risk in relatives of affected individuals indicate a genetic component in the disease's etiopathogenesis, but knowledge of the genetics of PEX is rudimentary. Specific mutations of the LOXL1 gene, a member of the lysyl oxidase gene family which plays a key role in elastin metabolism, are strongly associated with the development of PEX and PEXG [8].

To date, there have been several reports describing the presence of pseudoexfoliative material found on an IOL of one eye [1–5]. To our knowledge, our case would be the first to describe a bilateral finding of pseudoexfoliative material on IOLs of both eyes. The importance of this case is to raise awareness for the need of a thorough examination, even in pseudophakic patients, for it could be the only sign in the diagnosis of secondary open-angle glaucoma.

## Literature Search

A PubMed search was conducted, without date restriction, using the following terms: bilateral pseudoexfoliation deposits on intraocular lens implants, pseudoexfoliative syndrome, and pseudoexfoliative glaucoma.
